# Dietary fish oil (MaxEPA) enhances pancreatic carcinogenesis in azaserine-treated rats.

**DOI:** 10.1038/bjc.1996.7

**Published:** 1996-01

**Authors:** M. J. Appel, R. A. Woutersen

**Affiliations:** TNO Nutrition and Food Research Institute, Department of Pathology, Zeist, The Netherlands.

## Abstract

In the present study the putative chemopreventive effect of dietary fish oil (MaxEPA) on azaserine-induced pancreatic carcinogenesis in rats was investigated. Groups of rats were maintained on a semipurified low-fat (LF; 5 wt%) diet or on semipurified high-fat (HF; 25 wt%) diets containing 5 wt% linoleic acid (LA) and including 0.0, 1.2, 2.4, 4.7, 7.1 or 9.4 wt% MaxEPA. Animals fed a HF diet developed significantly higher mean numbers of atypical acinar cell nodules (AACNs), adenomas and carcinomas than animals fed a LF diet. Dietary MaxEPA caused a significant (P < 0.01) dose-related increase in mean number of AACNs (0.5 < phi < 3.0 mm). The mean number of adenomas and carcinomas remained similar among the groups. Cell proliferation was significantly lower in AACNs from animals fed HF containing 9.4% MaxEPA in comparison with HF without MaxEPA and with LF. LA levels had increased and arachidonic acid (AA) levels had decreased in blood plasma and pancreas with increasing dietary MaxEPA. Feeding MaxEPA resulted in significant decreases in 6-keto-prostaglandin (PG) F1 alpha (P < 0.05) and PGF2 alpha (P < 0.01) in non-tumorous pancreas, whereas PGE2, PGF2 alpha and thromboxane B2 (TXB2) levels were significantly (P < 0.001) higher in pancreatic tumour tissue than in non-tumorous pancreatic tissue. It is concluded that (i) dietary MaxEPA enhances dose-relatively growth of putative preneoplastic AACNs in the pancreas of azaserine-treated rats; (ii) dietary MaxEPA inhibits the conversion of LA to AA, as well as the conversion of AA to TXB2 or PGF2 alpha in non-tumorous pancreatic tissue; (iii) the high levels of PGE2, PGF2 alpha and TXB2 in pancreatic adenocarcinomas indicate a possible role for these eicosanoids in modulation of tumour growth.


					
British Journal of Cancer (1996) 73, 36-43

AA       ? 1996 Stockton Press All rights reserved 0007-0920/96 $12.00

Dietary fish oil (MaxEPA) enhances pancreatic carcinogenesis in
azaserine-treated rats

MJ Appel and RA Woutersen

TNO Nutrition and Food Research Institute, Toxicology Division, Department of Pathology, PO Box 360, 3700 AJ, Zeist, The
Netherlands.

Summary In the present study the putative chemopreventive effect of dietary fish oil (MaxEPA) on azaserine-
induced pancreatic carcinogenesis in rats was investigated. Groups of rats were maintained on a semipurified
low-fat (LF; 5 wt%) diet or on semipurified high-fat (HF; 25 wt%) diets containing 5 wt% linoleic acid (LA)
and including 0.0, 1.2, 2.4, 4.7, 7.1 or 9.4 wt% MaxEPA. Animals fed a HF diet developed significantly higher
mean numbers of atypical acinar cell nodules (AACNs), adenomas and carcinomas than animals fed a LF
diet. Dietary MaxEPA caused a significant (P<0.01) dose-related increase in mean number of AACNs
(0.5 <0 < 3.0 mm). The mean number of adenomas and carcinomas remained similar among the groups. Cell
proliferation was significantly lower in AACNs from animals fed HF containing 9.4% MaxEPA in com-
parison with HF without MaxEPA and with LF. LA levels had increased and arachidonic acid (AA) levels
had decreased in blood plasma and pancreas with increasing dietary MaxEPA. Feeding MaxEPA resulted in
significant decreases in 6-keto-prostaglandin (PG) Fl,, (P < 0.05) and PGF2, (P < 0.01) in non-tumorous
pancreas, whereas PGE2, PGF2,, and thromboxane B2 (TXB2) levels were significantly (P<0.001) higher in
pancreatic tumour tissue than in non-tumorous pancreatic tissue. It is concluded that (i) dietary MaxEPA
enhances dose-relatedly growth of putative preneoplastic AACNs in the pancreas of azaserine-treated rats; (ii)
dietary MaxEPA inhibits the conversion of LA to AA, as well as the conversion of AA to TXB2 or PGF2,, in
non-tumorous pancreatic tissue; (iii) the high levels of PGE2, PGF2,, and TXB2 in pancreatic adenocarcinomas
indicate a possible role for these eicosanoids in modulation of tumour growth.

Keywords: pancreatic carcinogenesis; rat; azaserine; fish oil; prostaglandins; cell proliferation

Polyunsaturated fatty acids (PUFAs) from the w-3 family
(abundant in fish oil) have been shown to inhibit tumour
development in animal models for mammary (Jurkowski and
Cave, 1985), colon (Reddy and Sugie, 1988) and pancreatic
carcinogenesis. Information on effects of fish oil on panc-
reatic carcinogenesis is scarce and comes mainly from the
studies of O'Connor et al. (1985), who observed that in
azaserine-treated rats maintained on a 20% menhaden oil
(MO) diet for 4 months, the number and size of pancreatic
preneoplastic atypical acinar cell nodules (AACNs) were
significantly reduced as compared with rats fed a 20% corn
oil (CO) diet. In a subsequent 4 month study they found a
decrease in the number of AACNs with increasing levels of
MO in a 20% fat diet (O'Connor et al., 1989). However,
using the same model, we did not find any difference in
AACN yield in rats fed 25% fat diets with a constant 5%
linoleic acid (LA) level, either or not containing 9.4% fish oil
(MaxEPA), for 6 months (Appel and Woutersen, 1994).
Because of the latter unexpected and contrasting result and
the lack of data on effects of fish oil on development of
pancreatic tumours, we performed a 12 month study to
investigate the effects of increasing levels of MaxEPA in a
25% fat/5% LA diet on pancreatic tumour development in
azaserine-treated rats. Furthermore, the effects of dietary
MaxEPA on cell proliferation in AACNs and normal acinar
pancreatic tissue, as well as fatty acid profiles and prosta-
glandin levels in pancreatic tissue were examined.

with wire-mesh floors and fronts and were fed a standard
laboratory chow. Two weeks (? 1 day) after arrival the rats
gave birth to a mean of eight pups. After 4 days the pups
were sexed. All females, the surplus of male pups and the
surplus of mothers were killed and a total of 210 male pups
were divided among the remaining 26 mothers. One hundred
and seventy-five pups were given an i.p. injection of 30 mg
azaserine (Calbiochem-Behring, La Jolla, CA, USA) per kg
body wt, which was dissolved freshly in 0.9% sodium
chloride solution, at 14 and 21 days of age. Thirty-five
control pups received injections with sodium chloride solu-
tion alone. Directly after the second injection the animals
were weaned and randomly allocated to seven groups of 30
animals each (five control animals and 25 azaserine-treated
animals). The animals were kept in stainless steel cages, with
wire-mesh floors and fronts, five animals per cage and under
standard laboratory conditions. One week after carcinogen

treatment the rats were fed an AIN76-based purified diet

containing either 5 or 25 wt% fat. The control group
received a S wt% lard (Best Food, The Netherlands; LF)
diet, containing a marginal (0.61 wt%) but sufficient level of
linoleic acid (LA; National Research Council Subcommittee,
1978). The experimental groups received a high-fat (25 wt%;
HF) diet containing 5 wt% LA and including 0.0, 1.2, 2.4,
4.7, 7.1 or 9.4 wt% (0, 2.5, 5, 10, 15 and 20 en%) MaxEPA.
The experimental design is summarised in Table I. The diets
were compounded by mixing high-linoleic safflower oil
(Unilever, Vlaardingen, The Netherlands) with high-oleic

Materials and methods

Animals and diets

Fifty-five 1 week pregnant female Wistar rats were obtained
from Harlan-CPB, Austerlitz, The Netherlands. During preg-
nancy the rats were kept solitary, in stainless-steel cages fitted

Table I Experimental groups and carcinogen treatment

Experimental groups

Low fat                High fat
Treatment      (5 wt %)               (25 wt %)

Wt%   MaxEPA       0.0    0.0    1.2   2.4   4.7   7.1   9.4
Wt% linoleic       0.6     5.0   5.0   5.0   5.0   5.0   5.0

acid

No. of rats

Saline            5       5     5     5     5     5     5
Azaserine        25      25    25    25    25    25    25

Correspondence: RA Woutersen

Received 22 February 1995; revised 30 May 1995; accepted 19 July
1995

sunflower oil (Trisun, Contined, Bennekom, The Nether-
lands) and fish oil (MaxEPA; Seven Seas, Hull, UK). The
safflower oil, the sunflower oil and the MaxEPA contained
0.55gkg- ', 0.44gkg- ' and 1.80gkg-' vitamin E respec-
tively. a-Tocopherol was added to all diets as extra antioxi-
dant to a level of 0.450 g kg-'. The composition of the
AIN76-based diets and the fatty acid composition of the oils
are summarised in Tables II and III. The diets were prepared
monthly and stored at -20?C until use. The animals were
fed daily to minimise oxidation of the polyunsaturated fatty
acids. Peroxide values (as measured by means of the AOCS
official method in terms of milliequivalents peroxide per kg)
of the HF/9.4% MaxEPA-containing diet, stored at -20?C
for 3 months, were below 1.0 and remained below 1.0 when
exposed to air at room temperature for 24 h. Longer periods
of exposure to air at room temperature caused a rapid
increase in the peroxide value. The profiles of the dietary
fatty acids of interest are depicted in Figure 1. Food con-
sumption was measured daily during the first 3 months and
on 7 consecutive days per month during the remainder of the
study. The animals were weighed weekly during the first 3
months of the study and monthly thereafter.

Monitoring and autopsy

Three days before autopsy five saline-treated control rats and
five azaserine-treated rats from the LF, HF/0.0% MaxEPA
and the HF/9.4% MaxEPA groups had an Alzet osmotic
pump (Alza, Palo Alto, USA, model 2001) implanted sub-
cutaneously, containing 200 jil of a bromodeoxyuridine
(BrdU) solution (Sigma, Brussels, Belgium; conc. 25 mg
ml-'). The release rate of this pump was 1 jl h-<. Autopsy
was performed 343, 344 or 345 days after the last injection of
azaserine. The animals were anaesthetised with ether and
exsanguinated by cannulating the abdominal aorta. Blood
was collected in heparin-containing tubes, centrifuged at
1700 g for 20 min and stored at - 80?C until analysis. The
pancreas and liver were excised and weighed. About one-
third of the pancreas of two animals per cage was snap

Table II Percentage fatty acid composition of the dietary lipids
Fatty acid                 Lard      SA       SO     MaxEPA
C14:0                       1.8      0.1       0.1      7.1
C16:0                      25.6      7.0       3.8     17.5
C16:1                       2.9      0.1       0.1      9.9
C18:0                      14.2      2.6       4.0      4.2
C18:1                      43.1     13.1      82.6     12.9
C18:2 (w-6)                 8.7     76.0       7.7      4.2
C18:3 (w-3)                 0.6      0.4       0.1      0.0
C20:0                       0.2      0.3       0.3      2.5
C20:1                       0.8      0.2       0.3      4.4
C20:4 (w-6)                 0.0      0.0       0.0      1.6
C20:5 (w-3)                 0.0      0.0       0.0     18.2
C22:0                       0.1      0.2       0.9      0.0
C22:1                       0.0      0.0       0.0      1.1
C22:4 (w6)                  0.0      0.0       0.0      1.2
C22:6 ()3)                  0.0      0.0       0.0     14.9
Total                      98.0     99.9     100.0     99.7
o-3/w-6 (ratio)             0.07     0.01      0.01     4.7

SA, Safflower oil; SO, Sunflower oil (Trisun).

Modulation of pancreatic carcinogenesis by dietary fish oil
MJ Appel and RA Woutersen

37
frozen in liquid nitrogen and stored at - 80?C until fatty acid
or prostaglandin analyses. The remaining two-thirds of these
pancreata plus all other pancreata and all livers were fixed in
10% neutral buffered formalin. Livers and pancreata of
BrdU-treated animals were fixed for 24h in formalin fol-
lowed by 72 h in 70% ethanol. The organs were processed
for microscopy by conventional methods, step-sectioned at
5 1im and collected on organosilane-coated slides. Parallel
sections were stained with haematoxylin and eosin (H&E) or
with a monoclonal antibody against BrdU (Organon Tech-
nics, the Netherlands) and examined by light microscopy. In
the H&E-stained slides all pancreatic lesions were identified
as acidophilic or basophilic atypical acinar cell foci (AACF),
localised carcinoma (carcinoma in situ; CIS) or invasive car-
cinomas according to the criteria of Longnecker (1983) and
Rao et al. (1982). Basophilic AACF were not scored because
of insufficient yield. The area of the acidophilic AACF was
determined by using an intraocular grid as described before
(Woutersen et al., 1986). The volumetric data were estimated
using the method of Campbell et al. (1982). In slides stained
for BrdU the labelling index (LI) was expressed as the
percentage of brown-stained BrdU-positive nuclei of the total
number of nuclei counted. To select a random sample of
acinar cells, only nuclei that were located beneath the cross-
ings of the horizontal and vertical lines in a 20 x 20 intra-
ocular grid at high-power magnification (400 x) were
counted. In normal pancreatic tissue at least 1000 nuclei per
pancreas were counted. A mean of 8.8 AACF per pancreas
was evaluated (without discrimination between focus size)
and a mean of 133 nuclei per AACF was counted.

Analytical procedures

Fatty acids Pancreatic microsomes were prepared by
homogenising 100 -200 mg of pancreatic tissue in 0.1 M Tris-
KCI buffer, pH 7.4. Subsequently, the homogenate was cent-
rifuged at 10 00 g for 30 min and the supernatant was cent-
rifuged at 105 000 g for 60 min. The microsomal pellet was
resuspended in 300 ,ul of buffer and stored at - 30?C until

66

00

. ..

u

4-

0)
a)

cJ

t)

L-

luu

90
80
70
60
50
40
30
20
10

0

3-

u)

"0

6 ,

5t

CD
a)

210 C

2 3  4  5  6  7  8  9  10

Weight % of dietary MaxEPA

Figure 1 Percentage of selected dietary fatty acids. 0, Linoleic
acid; 0, oleic acid; 0, eicosapentaenoic acid; *, docosahex-
aenoic acid.

Table III Weight percentage composition of the AIN76-based diets

Premix                                                            LF                     HF/wt % MaxEPA

Diet components                   LF        HF                             0.0%     1.2%     2.4%    4.7%     7.1%     9.4%

Casein                         20.00     25.00   Premix        95.00    75.00    75.00    75.00    75.00    75.00   75.00
DL-Methionine                   0.30      0.37   Lard           4.74     -        -        -        -        -       -

Wheat starch                   63.50     35.79   Safflower oil  0.26     4.63     4.67     4.69     4.80     4.91    5.04
Cellulose                       5.00      6.18   Sunflower oil  -       20.37    19.15    17.95    15.49    13.04   10.53
Choline bitartrate              0.20      0.25   MaxEPA         -        -        1.18     2.36     4.71     7.05    9.43
AIN76 minerals                  3.50      4.32
AIN76 vitamins                  1.00      1.24
Calcium dihydrogen phosphate    1.50       1.85

Total                            95.00     75.00   Total         100.00   100.00  100.00   100.00   100.00   100.00  100.00

inn -

-7

.i

10 _                    Modulation of pancreatic carcinogenesis by dietary fish oil

MJ Appel and RA Woutersen
38

fatty acid analysis. Total lipids were extracted from 50 pAl
aliquots of pancreatic microsomes or from 100 ,lI aliquots of
blood plasma as described by Folch et al. (1957). Fatty acid
composition was determined by gas-liquid chromatography.
The samples were eluted on a capillary BD23 column (J&W
Scientific) after saponification with sodium hydroxide in
methanol and transmethylation of the fatty acids with boron-
trifluoride in methanol.

Prostaglandins Pancreatic  tissue  (100-200 mg)  was
homogenised in 0.1 M phosphate-buffered saline (PBS;
pH 7.4) containing 15% methanol. Before, during and after
the homogenisation procedure the samples were kept on ice.
The samples were applied to Sep-pak C,8 columns (JT Baker,
Phillipsburg, NJ, USA), and, after washing with 6 ml of 15%
methanol/PBS and 6 ml of petroleum ether, eluted with 6 ml
of methanol. After evaporation of the methanol under nit-
rogen, the samples were dissolved in 1.0 ml of potassium
phosphate buffer (1.0 M; pH 7.4) and subsequently analysed
by using enzyme immunoassay kits for PGE2, PFE2C,, 6-keto-
PGFIc, and TXB2 (Cascade Biochem Ltd, Reading, UK).

Statistics Food and energy intake and body and pancreatic
weights were statistically evaluated by two-way analysis of
variance followed by Dunnett's test, prostaglandin levels
were evaluated by analysis of variance followed by Student's
t-test, the number of pancreatic lesions was evaluated by
two-sample t-test, or by one-way analysis of variance fol-
lowed by linear trend tests with orthogonal contrasts. The
number of tumour-bearing animals (incidence) was analysed
by Pearson X2-test. Fatty acid compositions were evaluated
by two-way analysis of variance using percentage of dietary
MaxEPA and carcinogen-treatment as factors, and by one-
way analysis of variance followed by linear trend tests with
orthogonal contrasts.

Results

Food consumption and body and organ weights

Mean food consumption of rats maintained on an LF diet
was significantly (P<0.001) higher than that in rats main-

Table IV  Food and energy consumptiona

Food              Energy
LF                            15.4?0.3***            238.7
HF/0.0% MaxEPA                12.6  0.2              248.2
HF/1.2% MaxEPA                12.1 ?0.2              238.4
HF/2.4% MaxEPA                12.1  0.2              238.4
HF/4.7% MaxEPA                12.8  0.3              252.2
HF/7.1% MaxEPA                12.9  0.3             254.1
HF/9.4% MaxEPA                12.4  0.2              244.3

aFood intake in g day-' per animal; energy intake in kJ day-'.
Statistics: analysis of variance, ***P<0.001.

tained on an HF diet. However, owing to a higher energy
content of the HF diet, mean caloric intake was similar
among LF and HF groups (Table IV).

Mean body weight gain over the study showed no
significant differences among the groups (Figure 2). Mean
terminal body weights of animals fed a HF diet were
significantly higher relative to animals fed a LF diet
(P<0.05; Table V). Azaserine treatment caused a consistent,
significant increase in both absolute and relative pancreas
weights in all groups (P<0.001) in comparison with saline-
treated controls. Both absolute and relative liver weights
were significantly higher in animals kept on a HF diet than
in LF controls (P<0.01 and P<0.05 respectively).

Microscopy

Feeding a HF diet significantly enhanced tumour growth in
comparison with the LF control diet, as reflected by a
significantly higher number of tumour-bearing animals
(P<0.001), number of AACNs (P<0.01) and total number
of carcinomas (P<0.05). Including MaxEPA in the HF diet
resulted in a significant dose-related linear increase in number
of AACNs with both a diameter of 0.5 -1.0 mm as well as a
diameter of 1.0-3.0 mm (P<0.01). A similar effect was seen
on the volume of pancreas occupied by AACNs (P<0.01).
No such effect was seen on the number of adenomas and
carcinomas or on the number of tumour-bearing animals
(Table VI and Figure 3).

Cell proliferation

Labelling index in normal acinar cells was low (below 1%,
Figure 4) and similar in all groups. The mean LI in AACNs

700 -

600 -

500

0)

. _

-o
0

0

40(
30(

20(
10c

10      20     30      40      50

60

Experimental weeks

Figure 2 Body weight gain of azaserine-treated rats maintained
on a low-fat diet or a high-fat diet containing increasing levels of
MaxEPA for 12 months. *, LF; V, HF/0.0% MaxEPA; 0,
HF/1.2% MaxEPA; A, HF/2.4% MaxEPA; *, HF/4.7% Max-
EPA; *, HF/7.1% MaxEPA; 0, HF/9.4% MaxEPA.

Table V Body and organ weight at autopsya

Absolute weight (g)                     Relative weight (g kg-')

Diet group                      n       Body wt8    Pancreas wt     Liver wtd   Pancreas wt'    Liver wtb
LF                   Sal        5       546?22       1.07?0.06      12.2?1.0     1.97?0.12     22.3?1.2

Aza       25       546  10      1.47  0.06    14.0+? 0.4    2.69  0.10    25.7 0.5
HF/0.0% MaxEPA       Sal        4       588  24      1.07  0.08     15.1 0.8     1.81 0.06     25.6 0.4

Aza       22       571?12       1.80?0.14      14.6?0.5     3.13?0.19     25.4?0.6
HF/1.2% MaxEPA       Sal        5       584  36      0.93  0.07     14.6 0.9     1.58  0.06    24.9 0.3

Aza       21       570  13      1.57?0.10      14.7?0.5     2.76?0.16     25.8?0.6
HF/2.4% MaxEPA       Sal        5       566 31       1.26?0.07      14.6 1.3     2.23 0.11     25.6  1.3

Aza       23       591  17      1.79  0.18     15.7 ? 0.6   2.98 ? 0.21   26.5 ? 0.5
HF/4.7% MaxEPA       Sal        4       632?41       1.32?0.20      16.3? 1.2    2.17?0.44     25.8?0.6

Aza       24       595?16       1.96?0.15      15.7?0.6     3.28?0.22     26.2?0.5
HF/7.1% MaxEPA       Sal        5       637?51       1.04?0.09     16.5? 1.5     1.68?0.22     25.9?0.8

Aza       23       613?14       1.78?0.11      16.4?0.5     2.87?0.15     26.7?0.5
HF/9.4% MaxEPA       Sal        4       643 53       1.04?0.11      18.8? 1.9    1.65?0.25     29.1 0.8

Aza       22       572  17      1.95?0.15      16.2?0.7     3.37?0.20     28.1?0.8
aValues are means+s.e.m.; bp<OO.S (LF vs HF); CP<0.001 (Sal vs Aza); dP<O.Ol (LF vs HF).
LF, low fat; HF, high fat; Sal, saline-treated; Aza, Azaserine-treated.

_- -   _-- -

0 *

* I A

- i 71L m- - -a- -0 MI

e  -1- A      .,    a

Modulation of pancreatic carcinogenesis by dietary fish oil
MJ Appel and RA Woutersen

from animals fed the LF, the HF and the HF diet including
9.4% MaxEPA was 28.4 ? 3.2, 24.8 ? 1.7 and 15.5 ? 1.2
respectively. Cell proliferation was significantly higher in
AACNs in comparison with normal tissue (P<0.001). A
high level of MaxEPA in the HF diet caused a significant
(P <0.05) decrease of the LI in AACNs in comparison with
AACNs from both the HF diet without MaxEPA and the
LF diet.

Fatty acid analyses

Fatty acid profiles of blood plasma and pancreatic mic-
rosomes are presented in Tables VII and VIII. Statistics are
only presented on oleic acid (OA; C1 8:1), linoleic acid (LA;
C18:2), arachidonic acid (AA; C20:4), eicosapentaenoic acid
(EPA; C20:5) and docosahexaenoic acid (DHA; C22:6).

In blood plasma the levels of AA (C20:4) and DHA
(C22:6) were significantly higher in saline-treated vs
azaserine-treated rats (P<0.001). In pancreatic microsomes
the level of OA (C18:1) was significantly higher (P<0.05)
and the levels of AA (C20:4), EPA (C20:5) and DHA
(C22 :6) were significantly lower (P <0.001, P <0.05 and
P<0.001 respectively) in saline-treated rats in comparison
with azaserine-treated rats.

In blood plasma and pancreas the levels of OA (C18:1)
decreased and the levels of EPA (C20:5) and DHA (C22:6)
increased, parallel to the dietary supply of these fatty acids.
Levels of LA (C18:2) increased and AA (C20:4) decreased in
both blood plasma and pancreas of saline- and azaserine-
treated rats. Significant linear increases were observed in
levels of LA (C18:2; P<0.001), EPA (C20:5; P<0.001) and
DHA (C22:6;P <0.001) and significant linear decreases were
observed in levels of OA (C18:1; P<0.01) and AA (C20:4;
P<0.001; except for C20:4 in pancreas of saline-treated rats:
P =0.926).

LA (C 18:2) levels in microsomes from tumours were
significantly lower in all diet groups measured (P <0.05, at
least), whereas AA (C20:4) levels were significantly elevated
in tumours from animals fed 2.4% and 4.7% MaxEPA in
comparison with non-tumorous pancreas from azaserine-
treated rats (P <0.01). No differences were found in EPA
and DHA profiles, except for the DHA level in tumours
from the group fed 2.4% MaxEPA, which was significantly
higher in comparison with non-tumorous tissues (P<0.001).
The profiles of LA (C1 8:2), AA (C20:4), EPA (C20:5) and
DHA (C22:6) in pancreatic tumours are depicted in Figure 5.

Prostglandins

Pancreatic 6-keto-PGF1,- and PGF2, levels were significantly
lower in saline-treated rats maintained on the HF diet con-
taining 4.7% and 9.4% MaxEPA, in comparison with saline-
treated rats maintained on the HF diet without MaxEPA
(P < 0.05 and P < 0.01, for the respective prostaglandins). In
azaserine-treated rats PGF20 levels were significantly lower
(P <0.05) in pancreata from animals fed 9.4% MaxEPA in
comparison with those from animals fed the HF diet without
MaxEPA. Pancreatic PGF20 and TXB2 levels were inversely
related to the percentage of dietary MaxEPA in both saline-
and azaserine-treated rats. Parts of all grossly visible panc-
reatic rat tumours were analysed for prostaglandins. Micros-
copic examination of the grossly visible pancreatic lesions
indicated that the number of adenocarcinomas in the group
given high fat without MaxEPA was high enough (n = 3) to
justify statistical analysis. PGE2, TXB2, PGF2O, but not 6-
keto-PGF1, levels in pancreatic adenocarcinomas were
significantly elevated (P <0.001) in comparison with non-
tumorous pancreas from azaserine-treated rats (Figure 6).

0

C-

Cl

C-

6
0o

-o

0.[L

.0

._
co

0

._

c0

0

0 -

._O

cOd-

CO

.-  o

E0

-id

"I

0.,

27

0~

0 0 0 0)   NO C ) c   C--  0)

o.~~~~~~~~~~C

00  I/D O   oo   0 0

0 , . 0+I +I+I +I +I+I +I

Cl 00000- N

Cl-~ ~ ~ ~~~1

ON1   0 -  -   r e 0

00   en  "  6  o

N  o N    oNo

r- C,  +I +I +I +I +I +I +I

a, _  O  o 00 -t m,

- N    ON  Cl  O  N  - 0 N

C> r- ~ ~ C

Cl  oN  od  -0   o -

- 0 Cl  Cl  rA  NO

0  C<  d+I +I +I +I +I  +I +I

*.  r, C-- O  N   e   00  00

-   T  N) N  NO  -o

C-;

o ' N O  o  o Cl  NCl

-  -       Cl

0U  O~  e  Cl oN r mO

0- .l Cl 0 Cl .

oN- oo o o

_  _    N~~C

0

o.
o>

0000 NO 0 N rn -

C N    C - o   ot

c-i r 6 6 66 4

C Nt +1 +1 +1 +1 +1 +1 +1

o 0 C   l ON   NO VCd -  N

ON  0 00  0  en  o  oN  ON

-     06 C    _-

cf  00  NO  C 0  cO

~6   6 6 6   6 -

C  +1 +1 +1 +1 +1 +1 +1

- NO 00 NO CO 0 t NO

N_   N O  - 0 0   N

6  6o 6o 6 o  o

CA
CCOO

00

0CCZ

M 0o

0

Discussion

The results of the present study demonstrated a strong
enhancing effect of HF (25 wt%) diet including 5 wt% LA
on pancreatic carcinogenesis in azaserine-treated rats.

c00

0

0 0

O. ?

C)0 v

0_._

too

0
O

.0-.

0

o

o .?0
0,)o c-.

c) C =

0o8
* 0 Y

#, o.
- Cl

$W 0

.= .

04

0-e

ciLo5

+1.

0 0r

OcO

0on >

0: Q

on ccsZ

.~ 0 0ca

;: Y

8)vc

'IT
CA,
44
z
:z
"lp
.1
?t

E?-
liz

Modulation of pancreatic carcinogenesis by dietary fish oil

MJ Appel and RA Woutersen
40

z
u

0.0 1.0 2.0 3.0 4.0 5.0 6.0 7.0 8.0 9.0 10.0

Weight % dietary MaxEPA

u,

0

E

0
c

a-.)

Figure 3 Mean number of (pre)neoplastic lesions (? s.e.m.) in
the pancreas of azaserine-treated rats maintained on a high fat
diet containing increasing levels of MaxEPA for 12 months.
Statistics: analysis of variance followed by linear trend tests
(orthogonal contrasts).  0 , AACN >0.5 mm;  *  AACN
1-3 mm; C0, carcinoma.

Moreover, including various amounts of fish oil (MaxEPA)
in the HF diet resulted in a significant dose-related linear
increase in the number of putative preneoplastic AACNs
with a diameter between 0.5 and 3.0 mm. It appeared that
long-chain PUFAs of the w-3 series from fish oil in the diet
are readily taken up by the rat, resulting in increased concen-
trations in both plasma and pancreas, concomitant with in-
creasing concentrations in the feed. The presence of MaxEPA
in the diet significantly decreased cell proliferation in AACNs
and influenced the metabolism of linoleic acid, arachidonic
acid and and hence prostaglandin synthesis substantially.

In a number of studies the chemopreventive effect of
dietary fish oil has been reported in experimental mammary
gland carcinogenesis (Jurkowski and Cave, 1985; Braden and
Carroll, 1986; Ip et al., 1986; Abou-El-Ela et al., 1989), colon
carcinogenesis (Minoura et al., 1988; Reddy and Maruyama,
1986; Reddy and Sugie, 1988) and pancreatic carcinogenesis
(O'Connor et al., 1985; 1989). To investigate the effects of
fish oil (containing high concentrations of w-3 fatty acids),
most investigators used mixtures of corn oil and fish oil or
fish oil as such. Most of the studies that had the intention of
varying o-3 fatty acids in the diet, had an experimental
design also resulting in a variation in dietary w-6 fatty acid
levels. Moreover, in those studies where fish oil was used as
sole lipid source, the level of w-6 fatty acid frequently was a
deficient one.

O'Connor et al. (1985) reported that 20% MO caused a
significant inhibition of the growth of azaserine-induced
AACNs in rat pancreas as compared with a 20% CO diet. In
a subsequent study (O'Connor et al., 1989), they varied the
w-3/w-6 ratio from 0.01 up to 7.0 by mixing CO with MO
and observed a significant decrease in development of AACN
with increasing ratio of w-3/w-6. They acknowledged that
their dietary regimen also implied a variation of dietary LA
from 0.6% up to 12.0%, which is within the range in which
growth of AACN is significantly increased (4.4-8.5%;
Roebuck et al., 1985). In a previous study (Appel et al.,
1994), we varied the dietary LA levels from 2.0% to 15.0%,
without influencing the chain length of the fatty acids, and
observed an inverse rather than a positive dose-response
relationship between LA and AACN development. In order
to minimise the number of variables and their possible con-
founding effects, it seems of paramount importance to keep
LA in the diet at a constant level when investigating the
effects of other variables such as dietary fish oil. In the
present study an increase of MaxEPA paralleled a decrease
of oleic acid (OA). OA has been reported to enhance the
growth of pancreatic AACNs (Khoo et al., 1991). However,
in this study pancreatic AACNs were absent in the group
treated with azaserine alone and in the other groups the yield

Figure 4 Labelling index of normal and preneoplastic pancreatic
tissue (AACN) in rats maintained on a 5 wt% fat AIN76-based
diet, a 25 wt% fat AIN76-based diet or a 25% fat AIN76-based
diet containing 9.4 wt% MaxEPA for 12 months. Statistics:
analysis of variance followed by Student's t-tests. *P<0.05. *,
Normal tissue; _, AACN.

of AACNs was unusually low for the experimental protocol
used (30 mg of azaserine per kg body wt at 14, 21 and 28
days of age, followed by a 6 month feeding period). In our
studies dietary OA either correlated negatively (present
results) or positively (Appel et al., 1994) with the develop-
ment of AACNs. Apart from the isolated study by Khoo et
al., OA has not been implicated as a promoter in car-
cinogenesis. Moreover, since OA cannot be metabolised to
LA or other long-chain PUFAs and prostaglandins, the
findings of Khoo et al. (1989) are hard to explain mechanis-
tically. In our view, the influence of OA on pancreatic car-
cinogenesis seems to be of minor importance.

Apart from the importance of an adequate study design,
the present results also emphasise that an enhancing effect on
growth of early stages in pancreatic carcinogenesis (AACNs),
does not necessarily lead to more carcinomas. Consequently,
a long-term (12-15 months) study is needed to establish the
modulating effects of dietary or other factors on the develop-
ment of the ultimate pancreatic tumours.

The observation that after 12 but not after 6 months
(Appel and Woutersen, 1994) an enhancing effect of fish oil
on the growth of AACNs is seen, suggests that fish oil
modulated pancreatic carcinogenesis in azaserine-treated rats
only after a rather long period of feeding. Moreover, it
cannot be excluded that prolonged feeding of diets high in
MaxEPA for over 12 months will also finally modulate
development of pancreatic carcinomas. The significantly
higher LI of the acidophilic AACNs vs normal acinar tissue,
as observed in the present study, is in agreement with the
previously observed high growth potential of these putative
preneoplastic lesions (Rao et al., 1982). Although MaxEPA
caused a dose-related increase in number of AACNs, the LI
of AACNs in the pancreas of animals maintained on the HF
diet including 9.4% MaxEPA was significantly lower than
the LI of AACNs present in the pancreas of rats fed the HF
diet without MaxEPA. This seemingly contradictory observa-
tion may be due to MaxEPA-induced differences in cell
turnover, possibly caused by decreased apoptotic cell death.
The relation between cell proliferation and programmed cell
death is currently under investigation at our Institute.

The main compositional changes in fatty acids in blood
plasma and pancreas as a result of increasing MaxEPA in the
diet were a decrease in OA (C1 8: 1) and increases in EPA
(C20:5) and DHA (C22:6). An increasing level of dietary
MaxEPA caused a shift in the ratio of EPA/DHA from equal
or higher than 1 towards an EPA/DHA ratio of equal or less
than 1, both in plasma and pancreas. An explanation for this
interesting observation may be formation of DHA from
EPA. This newly formed DHA accompanied by DHA from
the diet accumulates, resulting in higher DHA levels than
EPA levels in plasma and tissue (Mathias and Dupont,
1989). Furthermore, the presently observed fatty acid profiles

Modulation of pancreatic carcinogenesis by dietary fish oil

MJ Appel and RA Woutersen                                                   A%

41

+1  1+  1+1 ++I+1 +1+1 ++1 1++I +1 +1+1 +1

0t (N  0o en   t   i   0 0 0 0 -   N%0   -

+l +1 +1 +1 +l +1 +1 +1 +l +1 +l +l +1 +1 +1

0 I  0 -  eC   0- e   t  C 0   0   -  (N  (N

+1 +I+1 +1 +I+1 +1 +1+1+1 +1+1 +1+1 +1

-O   -  - -           -

0    0   'f   0 0 0 0 0 0 0 0 - 0" 0   e

66666666666o-oo

+l +1 +1 +1 +1 +1 +1 +1 +1 +1 +1 +1 +l +1 +1

) (  I  SC  0   0t  00  T  -   " -  i (  N  0  C  ) O

0      - 0 0   - 00N 6 6

0 c0i 0- e 0   0-000000- 0-

+1 +1 +1 +1 +l +1 +1 +1 +1 +l +1 +1 +l +l +1

-      -( N            (N_^ t o   0

0D   -    - 00    0 CD  0 0  00- (N
000000 00 0 000)C-00m

+l +1 +l +1 +1 +l +l +1 +l +1 +1 +1 +1 +1 +1

- -   It  N14 en        ( en -   N  O  (N "

0  (N  O-0  0 0 0 0 0 0 0O0  0  0-

+l +l +l +1 +1 +1 +l +1 +1 +1 +1 +1 +1 +1 +1

0  c00 c ; 6 600000-0

+1 +l +1 +1 +1 +1 +l +l +1 +1 +1 +1 +1 +1 +l

- W  a' en  N4 en  < 'I  -  en en N   e 0  0 (

0(N 00 ( 0(N0000       0-0-

O D   en -O 00 0> 0D 0D 0 0

+1+1+1+1+1+1+1+1+1+1+1+1+1+1+1
_  _  e   eN   (   0  "  -  c  e  oO   c  0  0-

-     -(N-            (N

+1+1 +1+I+1 +1 +1+1 +I+1 +1 +1+1+1 +1

(n 00       m en      0 000 It  0 " en  en 0CN 0

.   .   .   .   .   .   .   .   .   . . . . .

+l +l +1 +l +1 +1 +1 +1 +1 +1 +l +1 +1 +1 +1

-00N0-0O00000-00
O 6   O ^oo mo o o o oonOo

C))

S:         IC

..  .  .  .  .  .  .  .  .   ..  ..  ..  ..  ..  ..

t ? ? O 000 0000000       0 o o  o o

?(NV(N(N(NV(N(N(N U           V

C

04
0
0
0
0

CO
a)

'0

C.

0

8

S.
Co

CI-

0

CO
CO

a)

C.)
Cd

CO

)._

0

cO

CO

CO
0
C.
CO
3I

0

NQ

~a
+1

._

.~o

0a

~cO

04)-

C O
F00

CO00

_CO

C. Q

CO

a))-

0 r

C .

1.C

0
(N

0

CO

0

u

ao

0

C_

CO

a)

4-

C.

._

co
CO

ed

0

C._

0

a)
"0

).e

._O

4-

CO

b

._

0

"0
a)

o
uB

a)
4-
ct

a)
a)
CO

a)

CO

N

Co

"0

._

CO

"0

C-

D)

-4

'IT
01
0.4

6)  )-

OC)).

0000-N        0000

+l +1 +1 +l +l +l +l +1 +1 +1 +1 +1 +1 +l +1

In In SO 000 xO N N N N (N ) -_0 en

0  N  0 -  00  (N   (N - 0

+l +1 +1 +1 +l +l ++ +1 +1 +l +1 +1 +1 +l +1

NO 0 O en t 0o C T en _ o o0 r -

1- s

0 000 - en O e -0 - 0 (N 00 en -
6 6 66 6 C'i 6 6 6 6 6 666 6

+1+1+1+1+1+1+1+1+1+1+1+1+1+1+1

n   N,-  0  n   Nl O~ C el  N-  "~ C~  0~ WI

0 ~ - N O   00 0 ( N -- 0 0 0C)M,   n

+1+1 +1 +1 +I+I+1 +1 +1 + +I+I+I+1 +1

(N  0t  -  N O  C)  0  -  N-   0 0 0 O   e   0

06 .i . - .6    .5 .6  .   . . .

6.266.2cN6666ooooo

+l +1 +1 +l +l +l +l +1 +1 +1 +1 +1 +1 +1 +l

In   0D NE   IC   e   '.  00  en  (   It  0  S/  (N  -   N

m t -

+l ++ +1 +l +1 +1 +1 +l +1 +1 +1 +1 +1 +1 +1

.  o)~  (N  0%  0%  =  0o -e . C   (N Co   e  n

o '.o-o  ro  r .  2 6o  o c   -

=       en<   <  --

6.2666.2666666666

+l +1 +1 +1 +1 +1 +1 +l +1 +1 +1 +l +1 +l +1
In n O61 ofi M: l O-< Cl 'R  n d- C^ O

CD 'tr  00  C>  N-  0"  (N  - C  (N ) ' .0   C 0 % (

+1 +I+1 +1+1+1 +1 +I+1 +I+1 +I+I+1 +1

0     I" -  .00Da   I'0 '.  00  O   O 0 O   "  -
0  00 0 0 r ~ 0 - - 0 ( N 'r-

+l +1 +l +1 +1 +1 +1 +1 +1 +1 +1 +l +1 +l +1

en   N 00  en r " N  (N (N  If  (N ",   00 en  -

00000-0 .000. M" 0000000 (N

+l +1 +1 +1 +1 ++ +1 +1 +1 +1 +1 +1 +1 +1 +1

'.0-   Ub (N0  .00000    (N00

00000 '.0 00 0 I 00- 0C en  00

+l +1 +1 +1 +1 +1 +l +l +1 +1 +1 +1 +1 +1 +1
" t C "It l-t e DC r-N   en r- 1. = =O
0- e 000 - el 000000000

m 0- d- i)n-o?oo      -

0 N -       CD  0 (N  (N -000       n   00

+l +1 +l +l +l +l +1 +1 +1 +1 +1 +1 +l +1 +1

it    0 o en  en dt 0 / -     0 0    00 0 0

6   .  . 6   .   .   -.  .   .   .   .   .  0   - 0

. -

u3 . r, '

= 0 ' 00 00 000 0000 ( N o o o ID

?(N(__      N ((N(N(N(

QQQQQQVUZQQQU   Q...

_____________u

O\
t'

D

E

S.C

co

0
co

r-
0

a)

e0
CO

N

COd

C.

0

CI

co

C'

C)-

00

C.

cn

0
0

u)

CO

CIS

2

a)

CO

a)

CO

N

CO
0
0
0

0

a)
0

CO
CO

0
a)

a)
0

-

CO

>

CO
o

CA
cd

C).

0

CO C

C.

c '

CO6
Q

a)..
COC

C.

o "

=" cn

COG

* 0
o (N

~"CO
~CO

D o

CO

<8

0
l. 0
+cn

N"0

C).

a) -
O.)
cnO*

+lo

..CO)~
0 cn

C.

u:

.- - - - - - - - - - - - - - - I

Modulation of pancreatic carcinogenesis by dietary fish oil
'"                                                         MJ Appel and RA Woutersen
42

20

-0

4-

0)
a)

0)

0)
0O

15

10

5

0

2U

C18:2

'a

._

0

0)
m
a)

0)
C)
03)

a-

15

10

5

0

0.0 1.0 2.0 3.0 4.0 5.0 6.0 7.0 8.0 9.0 10.0

Weight % dietary MaxEPA

C20:4

.    *  .-  .   *    ---   *   .  a  .   --

0.0 1.0 2.0 3.0 4.0 5.0 6.0 7.0 8.0 9.0 10.0

Weight % dietary MaxEPA

4

-0
0)

.)_
4-
4-

m

0)
a)

3

2
1

0

0.0 1.0 2.0 3.0 4.0 5.0 6.0 7.0 8.0 9.0 10.0

Weight % dietary MaxEPA

C22:6

/ -

0.0 1.0 2.0 3.0 4.0 5.0 6.0 7.0 8.0 9.0 10.0

Weight % dietary MaxEPA

Figure 5 LA (C18:2), AA (C20:4), EPA (C20:5) and DHA (C22:6) levels in microsomes of pancreas tumours (0) and
non-tumorous pancreas (U) of rats maintained on a high-fat diet containing increasing levels of MaxEPA.

a

0)

U)

Un

an

7

E
cm

0.

0)

U)

(A

en

0

cn
0

E

0

E
0.

70
60
50
40
30
20
10

0

b

280

0)
240 '

U)

._

200

160E
120

80
40
0

PGE2    6K-PGFia   TXB2      PGF2a

Figure 6 Prostaglandin levels in pancreas of (a) saline-treated
rats and (b) azaserine-treated rats. Statistics: analysis of variance
followed by Student's t-test. *P<0.05, **P<0.01, ***P<0.001.
NS, not significant.

show that feeding MaxEPA causes a decrease of AA (C20:4)
levels in both blood plasma (saline- and azaserine-treated
rats) and pancreatic tissue (azaserine-treated rats), which can
be ascribed to either replacement of AA by EPA (Mathias
and Dupont, 1989) or to an inhibition of the LA-converting
enzyme 6'-desaturase by EPA and/or DHA (Garg et al.,
1988). Evidence in favour of the latter process is given by the

observed increase in plasma and pancreatic levels of LA in
MaxEPA fed animals, although the amount of LA in the diet
was kept constant.

The differences between fatty acid profiles in pancreatic
tumours and non-tumorous tissue indicate an accelerated
conversion of LA to AA in tumour tissue, which is reflected
in consistently lower LA levels accompanied by higher AA
levels, pointing to an altered fatty acid metabolism in neop-
lastic tissue.

LA may give rise to PGs of the 2-series via AA. It has
been demonstrated that some of these PGs stimulate cell
proliferation (PGF1,,; Jimenez de Asua et al., 1983) or have
immunosuppressive properties (PGE2; Plescia and Racis,
1988) and hence may either promote tumour growth or
disturb inhibition of tumour development. w-3 PUFAs may
inhibit the formation of PGs derived from LA and AA, by
either inhibiting the conversion of LA to AA or inhibiting
the actual PG formation via cyclo-oxygenase. The dose-
related decrease of TXB2 and PGFa, in non-tumorous panc-
reatic tissue of rats maintained on HF diet containing 0.0%,
4.7% or 9.4% MaxEPA, together with the increased LA
levels in plasma and non-tumorous pancreas, demonstrate
that c-3 fatty acid influence LA/AA metabolism as expected.
The decreased PG-levels in AACN-containing pancreatic tis-
sue do not correlate with the increased mean number of
AACNs when dietary MaxEPA is increased, suggesting no
causal relationship between growth of preneoplastic acinar
lesions and prostaglandins of the 2-series. PG levels are
elevated, however, in pancreatic carcinomas indicating that
they may play a role in the development of AACNs to
ultimate pancreatic carcinomas.

From the present results it can be concluded that within
the duration of this study (i) dietary MaxEPA has a dose-
related enhancing effect on the development of AACNs but
not on development of carcinomas in the pancreas of
azaserine-treated rats and (ii) dietary MaxEPA inhibits the
conversion of LA to AA, as well as the conversion of AA to
TXB2 or PGF2, in non-tumorous pancreatic tissue and (iii)
PGs may play a role in the growth/development of panc-
reatic adenocarcinomas, but not in the growth of AACNs.

4

3
2

.)_

-0
C.)

4-
4_

m
0)

0)

0

1) A

r

1c _.

Zb I

r

I

F

Modulation of pancreatic carcinogenesis by dietary fish oil

MJ Appel and RA Woutersen                                                        w

43

Abbreviations

LA, linoleic acid; AA, arachidonic acid; OA, oleic acid; EPA,
eicosapentaenoic acid; DHA, docosahexaenoic acid; AACN, atypical
acinar cell nodule; AACF, atypical acinar cell focus; PG, prostaglan-
din; TXB2, thromboxane B2; PUFA, polyunsaturated fatty acid; CO,
corn oil; MO, menhaden oil; LI, labelling index.
Acknowledgements

We would like to thank Ms T Romijn for technical assistance, E
Schoen and J Hagenaars for their help with the statistical evaluation

of the data, Mrs A van Garderen-Hoetmer for her help with the
microscopic analysis of the pancreatic slides and Unilever Research,
Vlaardingen, for the kind gift of the safflower oil. We are grateful to
Professor VJ Feron and Professor RJJ Hermus for evaluation of the
draft. This work was supported by a grant from the Dutch Cancer
Society, CIVO 90-2.

References

ABOU-EL-ELA SH, PRASSE KW, FARRELL RL, CARROLL RW,

WADE AE AND BUNCE OP. (1989). Effects of D,L-2-
Difluoromethylornithine and indomethacin on mammary tumor
promotion in rats fed high n-3 and/or n-6 fat diets. Cancer Res.,
49, 1434-1440.

APPEL MJ AND WOUTERSEN RA. (1994). Modulation of growth and

cell turnover of preneoplastic lesions and of prostaglandin levels
in rat pancreas by dietary fish oil. Carcinogenesis, 15, 2107-2112.
APPEL MJ, VAN GARDEREN-HOETMER A AND WOUTERSEN RA.

(1994). Effects of dietary linoleic acid on pancreatic car-
cinogenesis in rats and hamsters. Cancer Res., 54, 2113-2120.
BRADEN LM AND CARROLL KK. (1986). Dietary polyunsaturated

fat in relation to mammary carcinogenesis in rats. Lipids, 21,
285-288.

CAMPBELL HA, PITOT HC, VAN POTTER R AND LAISHES BA.

(1982). Application of quantitative stereology to the evaluation of
enzyme-altered foci in rat liver. Cancer Res., 42, 465-472.

FOLCH J, LEES M AND SLOANE-STANLEY GH. (1957). A simple

method for the isolation and purification of total lipids from
animal tissue. J. Biol. Chem., 226, 497-509.

GARG ML, SEBOKOVA E, THOMSON ABR AND CLANDININ MT.

(1988). 66-Desaturase activity in liver microsomes of rats fed diets
enriched with cholesterol and/or w-3 fatty acids. Biochem. J., 249,
351-356.

IP C, IP MM AND SYLVESTER P. (1986). Relevance of trans fatty

acids and fish oil in animal tumorigenesis studies. In: Dietary Fat
and Cancer, Ip C, Birt DF, Rogers AE and Mettlin C. (eds).
Prog. Clin. Biol. Res. Series Vol. 222, pp. 283-294. Alan R. Liss:
New York.

JIMENEZ DE ASUA L, OTTO AM, LINDGREN JA AND HAMMARS-

TROM S. (1983). The stimulation of the initiation of DNA syn-
thesis and cell division in Swiss mouse 3T3 cells by prostaglandin
F2., requires specific functional groups in the molecule. J. Biol.
Chem., 258, 8774-8780.

JURKOWSKI JJ AND CAVE WT. (1985). Dietary effects of menhaden

oil on the growth and membrane lipid composition of rat mam-
mary tumours. J. Natl Cancer Inst., 74, 1145-1150.

KHOO DE, FLAKS B, OZTAS H, WILLIAMSON RCN AND HABIB NA.

(1991). Effects of dietary fatty acids on the early stages of neop-
lastic induction in the rat pancreas. Changes in fatty acid com-
position and development of atypical acinar cell nodules. Int. J.
Exp. Pathol., 72, 571-580.

LONGNECKER DS. (1983). Early morphologic markers for

carinogenicity in rat pancreas. In Application of Biological
Markers to Carcinogen Testing, Milman HA and Sell S. (eds)
pp. 43-60. Plenum Press: New York.

MATHIAS MM AND DUPONT J. (1989). Effects of dietary fat on

eicosanoid production in normal tissues. In: Carcinogenesis and
Dietary Fat. Abraham S. (ed.) pp. 29-51. Kluwer Academic Pub-
lishers: Boston.

MINOURA T, TAKATA T, SAKAGUCHI M, TAKADA H,

YAMAMURA M, HIOKI K AND YAMAMOTO M. (1988). Effect of
dietary eicosapentaenoic acid on azoxymethane-induced colon
carcinogenesis in rats. Cancer Res., 48, 4790-4794.

NATIONAL     RESEARCH     COUNCIL     SUBCOMMITTEE      ON

LABORATORY     ANIMAL     NUTRITION.    (1978).  Nutrient
Requirements of Domestic Animals. 10, 8-10.

O'CONNOR TP, ROEBUCK BD, PETERSON FJ AND CAMPBELL TC.

(1985). Effect of dietary intake of fish oil and fish protein on the
development of L-azaserine-induced preneoplastic lesions in the
rat pancreas. J. Natl Cancer Inst., 75, 959-962.

O'CONNOR TP, ROEBUCK BD, PETERSON FJ, LOKESH B, KINSELLA

JE AND CAMPBELL TC. (1989). Effect of dietary omega-3 and
omega-6 fatty acids on development of azaserine-induced
preneoplastic lesions in rat pancreas. J. Natl. Cancer Inst., 81,
858-863.

PLESCIA OJ AND RACIS S. (1988). Prostaglandins as physiological

immunoregulators. Prog. Allergy, 44, 153-171.

RAO MS, UPTON MP, SUBBARO V AND SCARPELLI DG. (1982).

Two populations of cells with differing proliferative capacities in
atypical acinar cell foci induced by 4-hydroxyaminoquinoline-l-
oxide in rat pancreas. Lab. Invest., 46, 527-534.

REDDY BS AND MARUYAMA H. (1986). Effect of dietary fish oil on

azoxymethane-induced colon carcinogenesis in male F344 rats.
Cancer Res., 46, 3367-3370.

REDDY BS AND SUGIE S. (1988). Effect of different levels of omega-

3 and omega-6 fatty acids on azoxymethane-induced colon car-
cinogenesis in F344 rats. Cancer Res., 48, 6642-6647.

ROEBUCK BD, LONGNECKER DS, BAUMGARTNER KJ AND

THRON DC. (1985). Carcinogen-induced lesions in the rat panc-
reas: effects of varying levels of essential fatty acid. Cancer Res.,
45, 5252-5256.

WOUTERSEN RA, VAN GARDEREN-HOETMER A, BAX J, FERINGA

AW AND SCHERER E. (1986). Modulation of putative prene-
oplastic foci in exocrine pancreas of rats and hamsters. I. Interac-
tion of dietary fat and ethanol. Carcinogenesis, 7, 1587-1593.

				


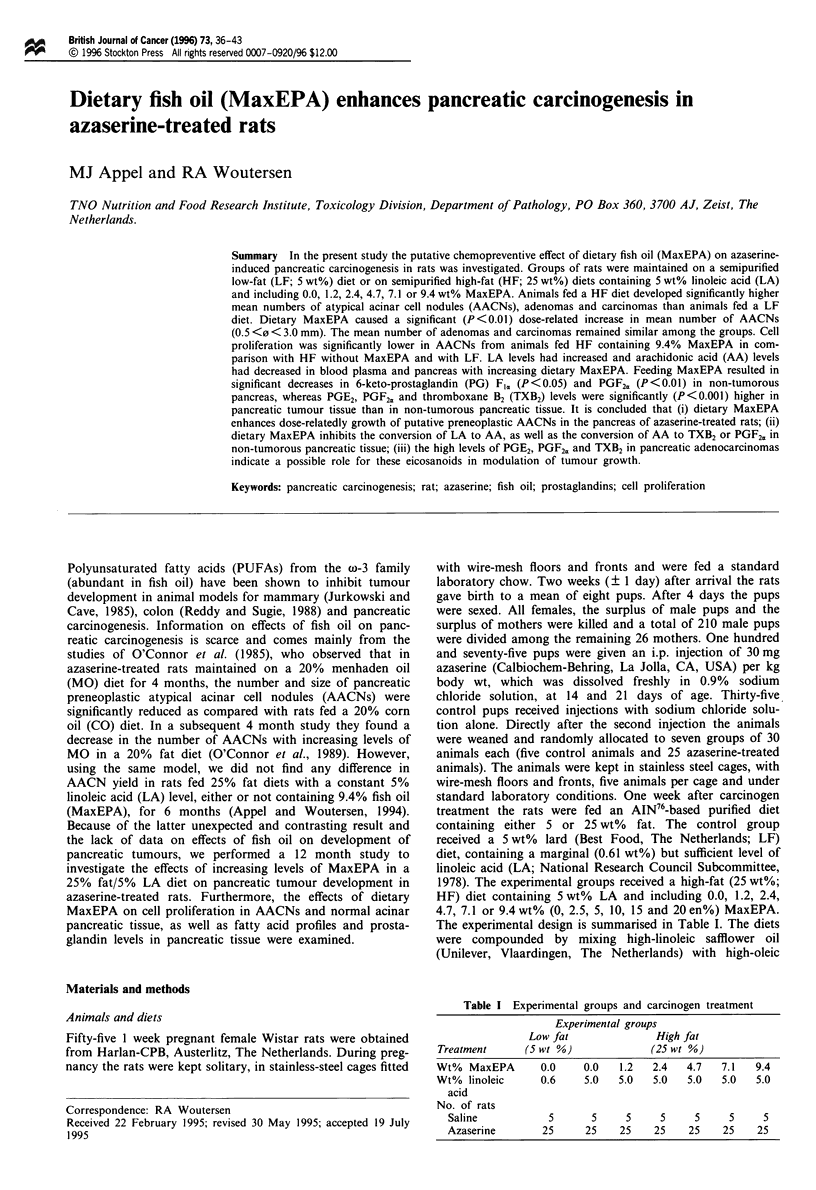

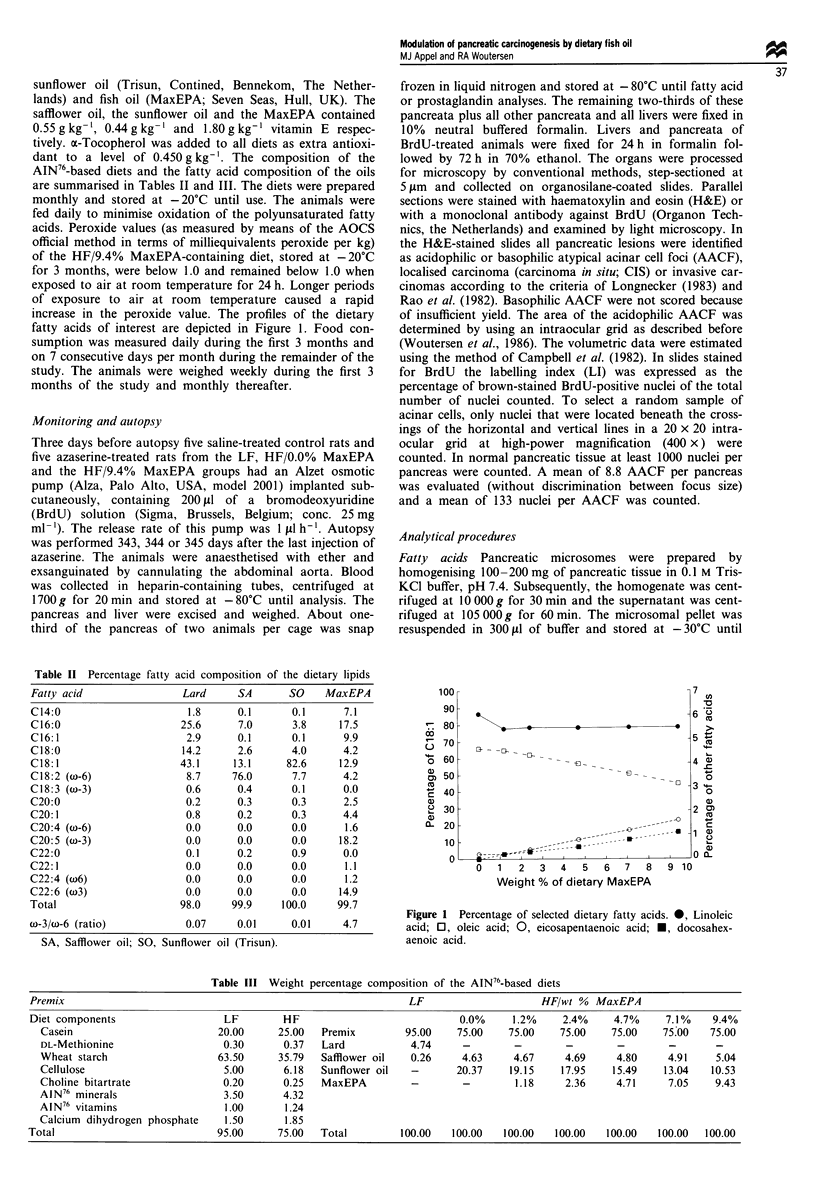

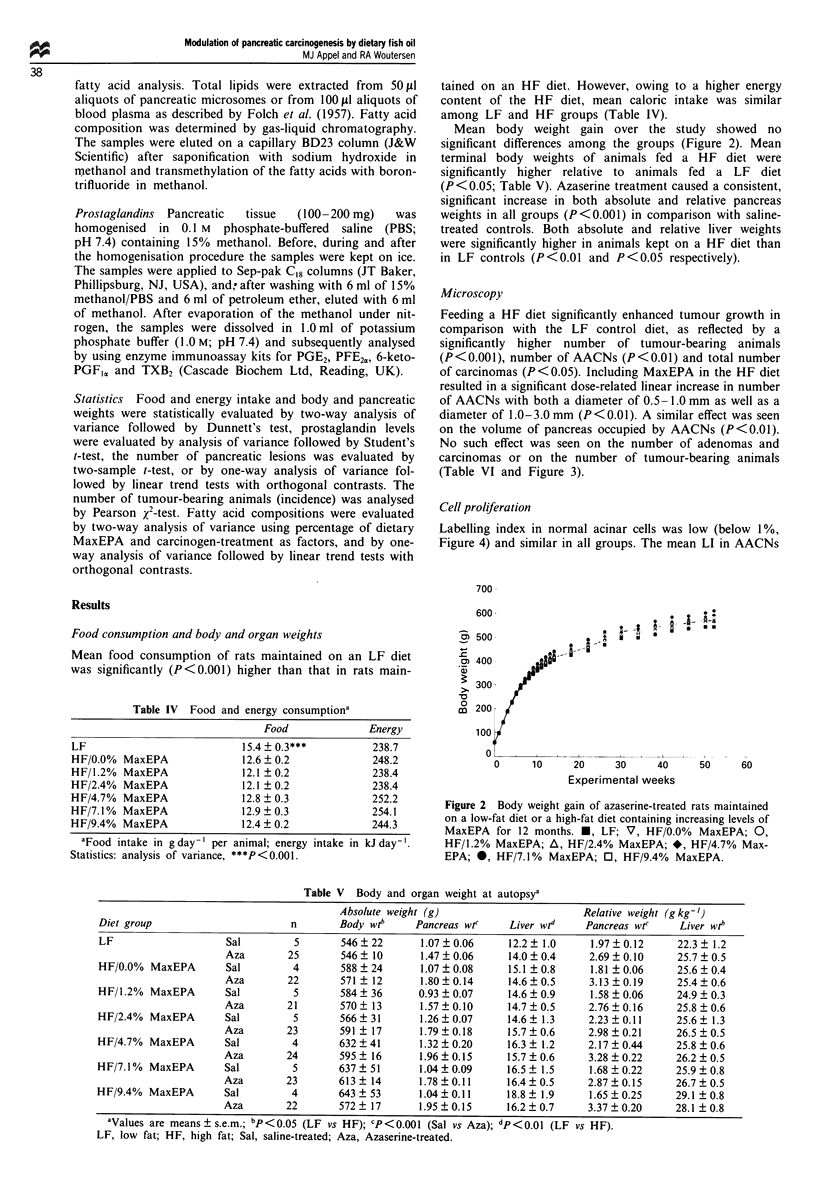

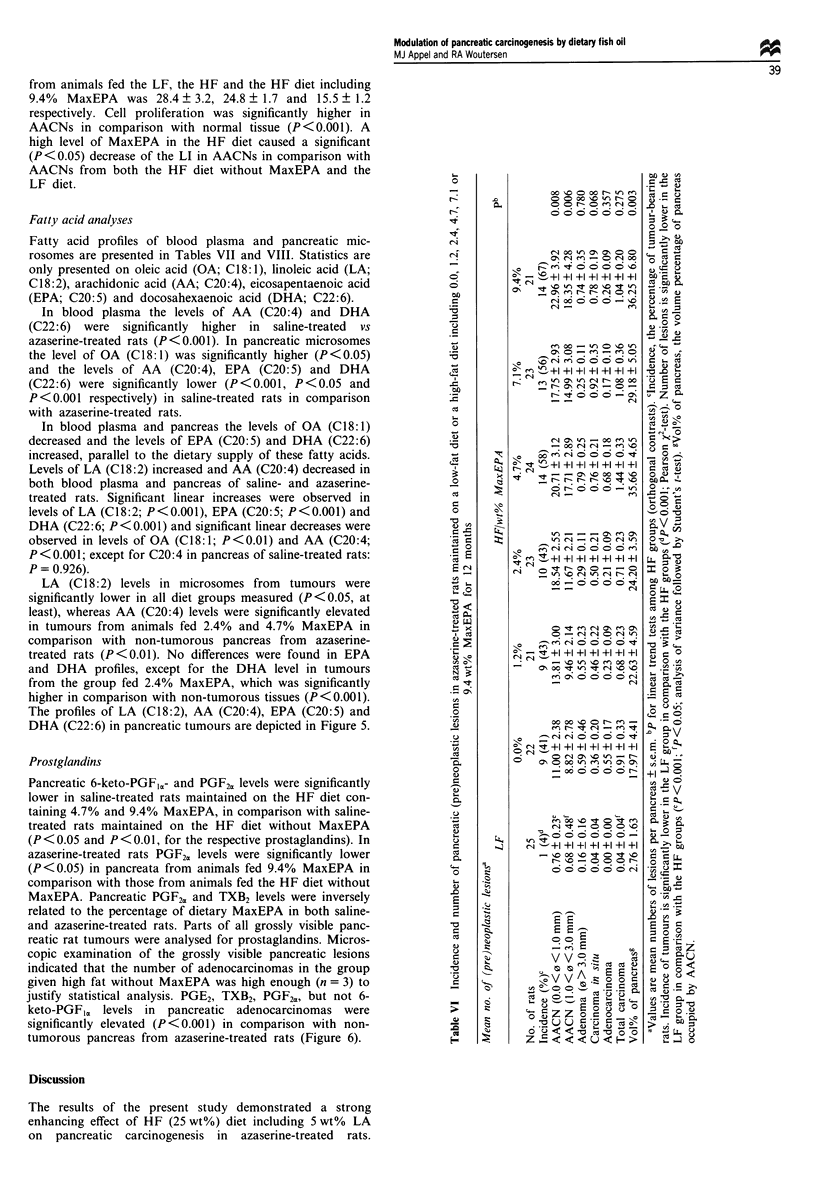

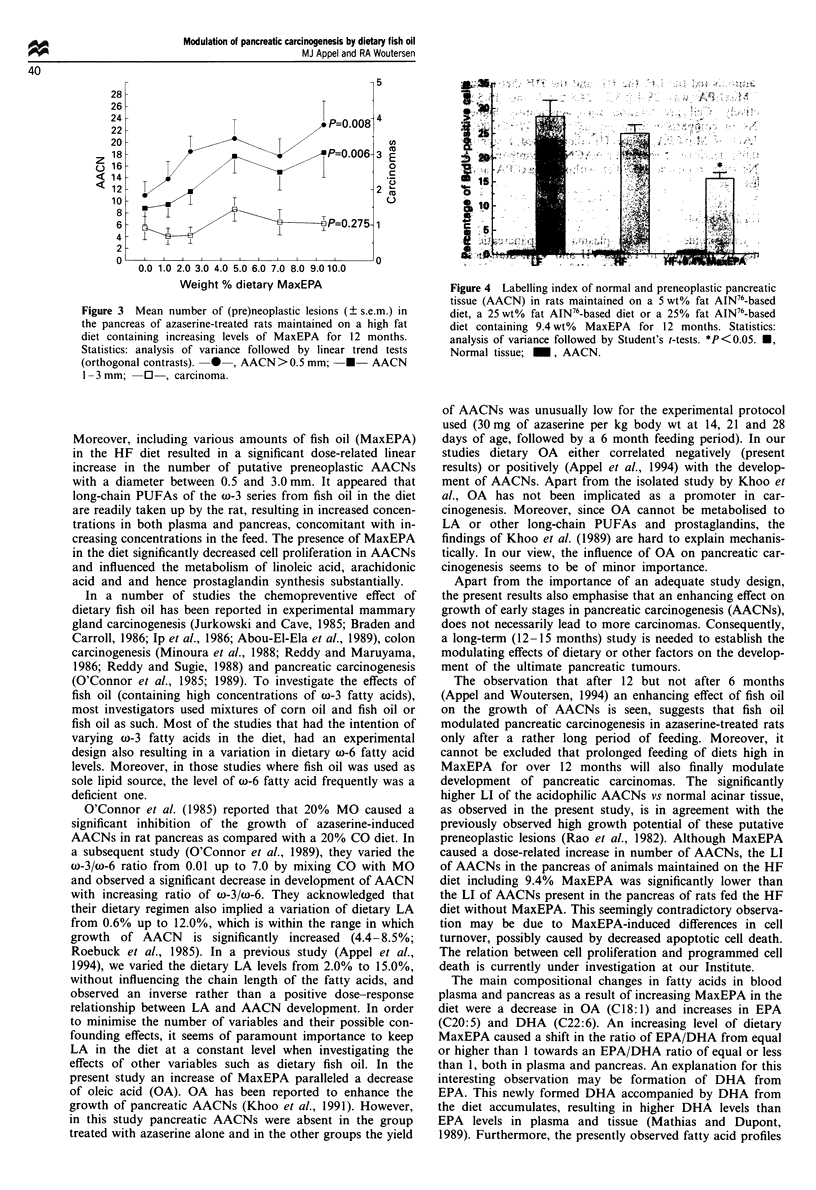

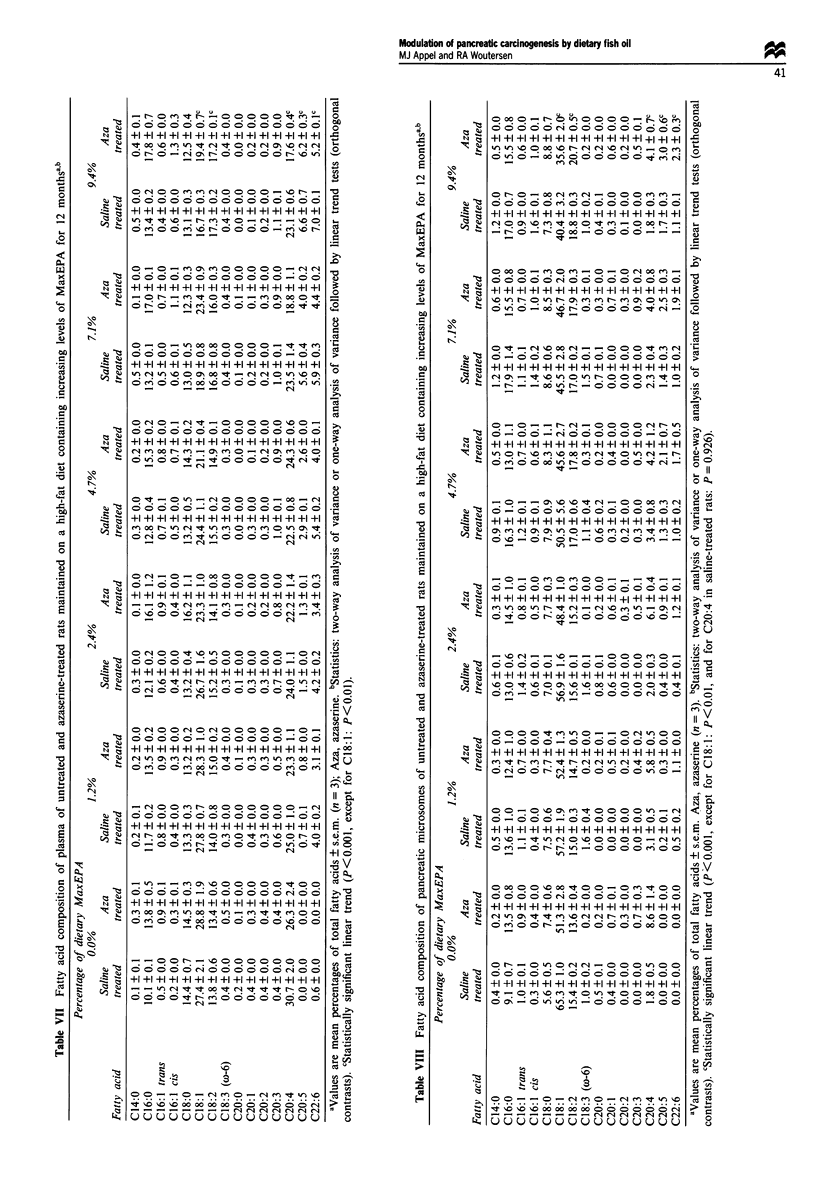

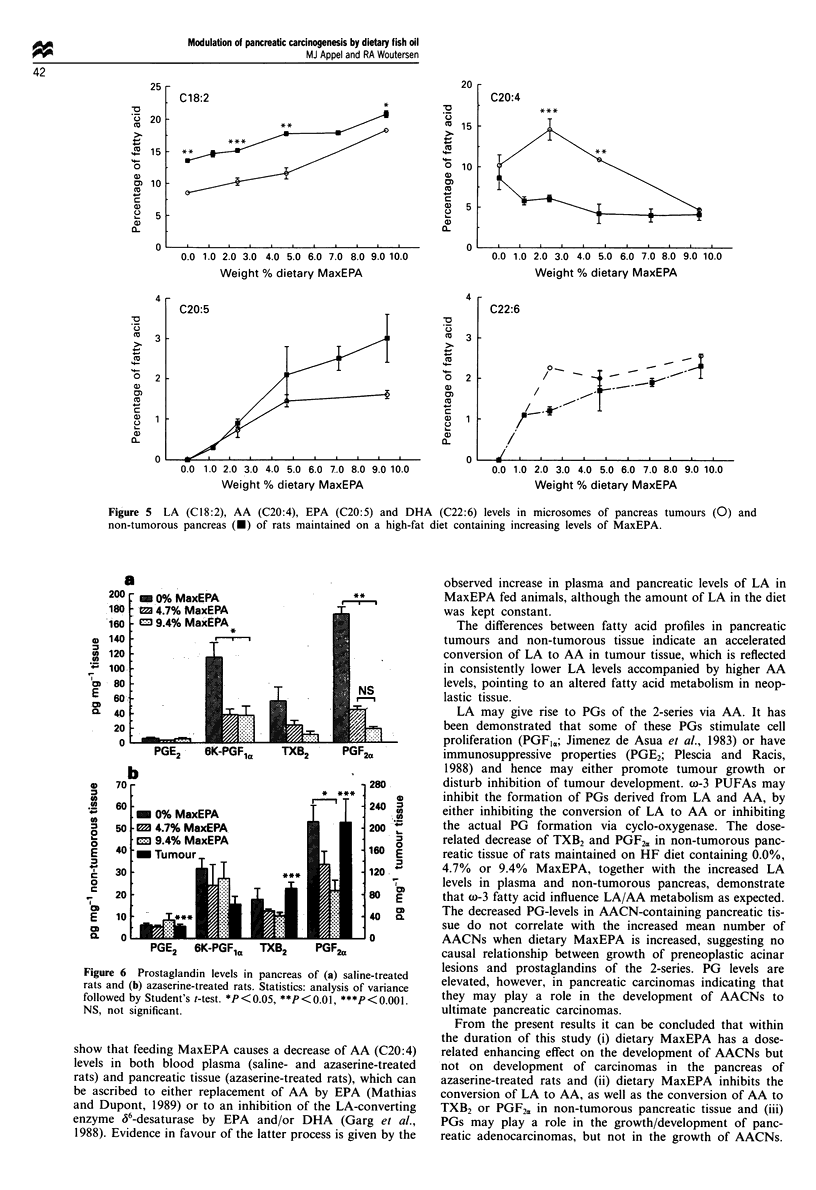

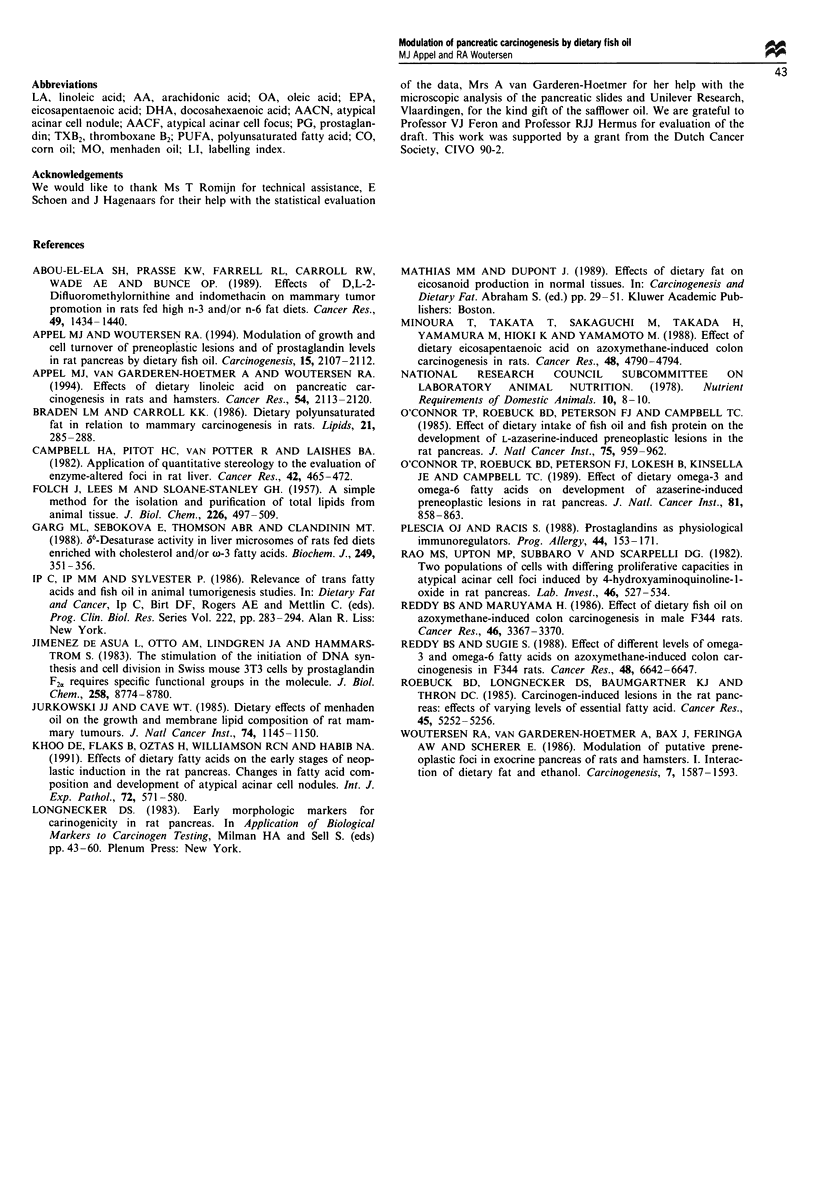

